# Micromechanical Characterization of Hydrogels Undergoing Swelling and Dissolution at Alkaline pH

**DOI:** 10.3390/gels3040044

**Published:** 2017-11-18

**Authors:** Wei Hu, Francois Martin, Romain Jeantet, Xiao Dong Chen, Ruben Mercadé-Prieto

**Affiliations:** 1 Suzhou Key Laboratory of Green Chemical Engineering, School of Chemical and Environmental Engineering, College of Chemistry, Chemical Engineering and Materials Science, Soochow University, Suzhou 215123, China; wei_hu_gem@163.com (W.H.); xdchen@mail.suda.edu.cn (X.D.C.); 2 Science et Technologie du Lait et de l’Oeuf (STLO), Agrocampus Ouest, INRA, 35000 Rennes, France; francois.martin@agrocampus-ouest.fr (F.M.); rjeantet@agrocampus-ouest.fr (R.J.)

**Keywords:** whey protein, indentation, shear modulus, swelling, dissolution

## Abstract

The swelling of polyelectrolyte hydrogels usually depends on the pH, and if the pH is high enough degradation can occur. A microindentation device was developed to dynamically test these processes in whey protein isolate hydrogels at alkaline pH 7–14. At low alkaline pH the shear modulus decreases during swelling, consistent with rubber elasticity theory, yet when chemical degradation occurs at pH ≥ 11.5 the modulus decreases quickly and extensively. The apparent modulus was constant with the indentation depth when swelling predominates, but gradients were observed when fast chemical degradation occurs at 0.05–0.1 M NaOH. In addition, these profiles were constant with time when dissolution rates are also constant, the first evidence that a swollen layer with steady state mechanical properties is achieved despite extensive dissolution. At >0.5 M NaOH, we provide mechanical evidence showing that most interactions inside the gels are destroyed, gels were very weak and hardly swell, yet they still dissolve very slowly. Microindentation can provide complementary valuable information to study the degradation of hydrogels.

## 1. Introduction

Protein-rich fouling deposits are commonly removed in the food industry using cleaning-in-place (CIP) systems using alkaline-based solutions at high temperatures, and usually at Reynolds number values over 70,000 [1]. Cleaning involves a chemical aspect, that is, alkali destroys chemical interactions between proteins, making the deposits weaker. In addition, extensive swelling can occur at extreme pH, low or high, due to the increase in the protein charge, common to many polyelectrolyte hydrogels [2], and also makes the deposits extremely weak. Cleaning also involves a mechanical component, which can be inferred from the enhancement of cleaning rates at higher fluid velocities [3]. It is expected that as deposits swell and are chemically degraded by the alkali, they become easier to be eroded by the flow. The removal of protein deposits on surfaces has been studied using many different techniques. For example, fluid dynamic gauging (FDG) can provide direct measurements of the deposit thickness [4], during swelling and dissolution, as well as the rupture shear of the soft material [5,6]; other studies simply quantify the concentration of proteins in solution to determine cleaning rates [3].

It is reasonable to suggest that the mechanical aspect of cleaning will involve the fluid conditions (e.g., velocity) as well as the properties of the deposit. However, scarce information is currently available on the dynamic mechanical or rupture properties of protein deposits undergoing swelling or dissolution, in particular, the important swollen layer next to the gel-solution boundary [7]. In order to obtain mechanical information of this swollen gel layer direct testing is required, ideally without damaging the deposit [8]. For this purpose, we have developed a micromanipulation technique capable of testing ad hoc hydrogels, which simulate hard-to-reproduce fouling deposits, in contact with very alkaline solutions, in situ and in real time. Testing was performed by indentation using a flat cylindrical punch. We report here an initial study showing the experimental capabilities of microindentation for the study of reactive swelling and dissolution, a very complex problem also present in many other soft degradable hydrogels [9,10].

## 2. Materials and Methods

### 2.1. Whey Protein Hydrogels

Heat-induced hydrogels were formed similarly to those reported previously [11] using commercial whey protein isolate (WPI) powder (BiPro, Davisco, La Crosse, WI, USA), with a protein content about 93 wt %. Solutions of 15 wt % WPI at pH ~7, with 0.1% sodium azide, were heated inside plastic test tubes for 35 min at 80 °C. These gels were stranded-like and fairly transparent, and were used unless mentioned otherwise. For comparison, particulate and fully opaque gels were also formed at 0.1 M NaCl; other gelation conditions were kept constant. Gels were stored overnight at 4 °C prior to being cut in cylinders of 5 ± 0.5 mm in height and 11 ± 0.5 mm in diameter. Gels were then submerged in solutions made at different NaOH and NaCl concentrations.

### 2.2. Dynamic Indentation Testing

**Figure 1:**
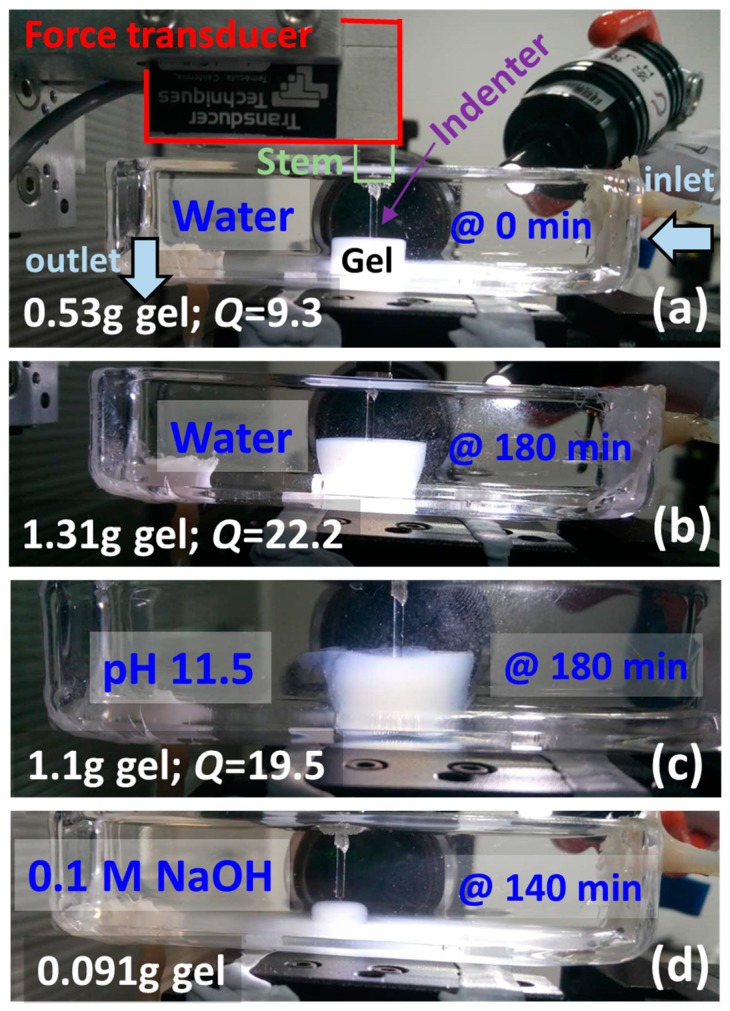
Experimental setup for the dynamic mechanical characterization of hydrogels by indentation. (**a**) Right after the gel is immersed in water, and (**b**) after 180 min; (**c**) in pH 11.5 after 180 min and (**d**) in 0.1 M NaOH after 140 min. The weight of the gels, as well as the calculated overall volumetric swelling degree *Q*, is also shown. Note that there is extensive dissolution in (**d**).

A new microindenter apparatus was designed to test soft materials. Vertical movements (e.g., indentations) were controlled by a nanostage (PPS-28-13300, Micronix, Santa Ana, CA, USA) with a digital encoder (MMC-100, Micronix), achieving a typical resolution of 2 nm, repeatability of ±20 nm, and accuracy of ±1 μm. The nanostage is positioned vertically with a force transducer connected using a custom-made holder. The force transducer used (GSO-10, Transducer Techniques, Temecula, CA, USA) has a nominal maximum load of 10 g. The signal of the force transducer was amplified (TMO-1, Transducer Techniques), and recorded at 20 Hz using LabView (and a USB-6002 datacard, National Instruments, Austin, TX, USA). Data was smoothed with a first-order Savitzky-Golay filter. A removable load stem (ALS-04, Transducer Techniques) was screwed in the force transducer. Glass rods of different sizes were inserted and glued to the stem. In this initial study, an indenter 1 mm in diameter was chosen as it was small enough to test at different locations of the gels, while being large enough to provide a good signal when the gels become very weak after swelling extensively. Flat and perpendicular glass surfaces were achieved after grinding (EG-45, Narishge, Tokyo, Japan). Hydrogels were placed inside a container, on top of a motorized XY stage where the desired solution was recirculated at 1 mL/min, as shown in Figure 1. Three consecutive loading-unloading indentations at 60 μm/s were made in different locations.

### 2.3. Data Analysis

The force at an indentation depth *h* using a flat punch indenter in an elastic sample is [12,13]:

(1) $$F_{E}=8GRh\Pi \left(R/t_{0},\nu_{P}\right)$$

where *G* is the instantaneous shear modulus in a linear elastic material; *R* is the radius of the indenter, *ν_P_* is the instantaneous Poisson’s ratio (0.477 for whey protein gels [14]), and Π(*R/t*_0_*,ν_P_*) is a dimensionless function needed to correct substrate effects, important for ratios of the indenter radius *R* to the initial sample thickness *t*_0_ (*R*/*t*_0_) > 0.1 [15].

Meanwhile Equation (1) is only valid for homogeneous samples, hydrogels undergoing swelling/dissolution are clearly not homogeneous, particularly close to the gel interface [16]. Hence, the *G* calculated represents some average value of the swollen layer tested. The reverse engineering problem whereby the depth dependence of the modulus is estimated from a single static loading experiment is very complex, if possible at all [17]. Techniques such as continuous stiffness measurements (CSM) [18] could be used in the future to obtain real estimates of *G* at different depths. In the meantime, static loading measurements will have to suffice to estimate qualitatively the mechanical inhomogeneity of the swollen layer.

**Figure 2:**
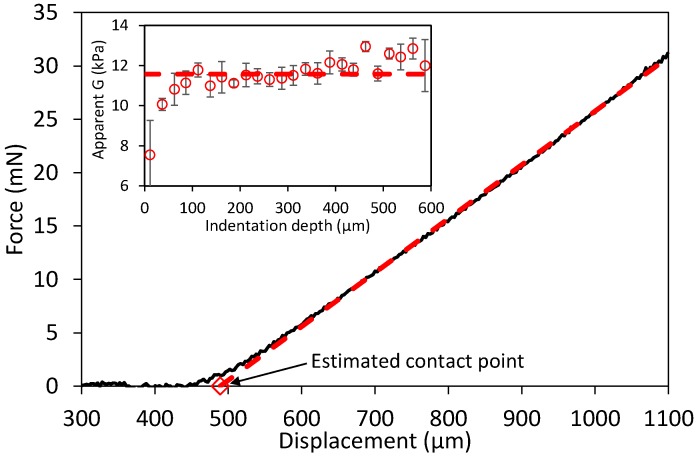
An example of an indentation of a whey protein isolate (WPI) hydrogel swollen from 3 min in water. The contact point is estimated by robust regression at large force values (dashed line), e.g., 6–31 mN here. The apparent shear modulus is then calculated using Equation (1) at different indentation depths at 25 μm intervals, shown in the inset, only for the purpose of checking the mechanical homogeneity of the indented layer. Error bars show the 95% CI of the fitted G. The dashed line in the inset is the average *G* between 50–500 μm.

In order to test the homogeneity of the hydrogels, indentation measurements were regressed at different indentation depths, usually at 25 μm intervals; an example is shown in Figure 2. Gels were indented typically up to 500–1000 μm, but only indentation data below 500 μm were considered in order for the linear elastic theory to be valid, typically at *h*/*t*_0_ < 0.1, where strains are assumed to be small and directly proportional to stresses [19]. The inset image in Figure 2 shows a representative example of constant shear moduli at different indentation depths. Corrections in the “toe region” due to imperfect contact, as commonly applied in homogeneous elastic materials [20], where not applied because it can also be due to swelling close to the boundary. For this reason, *G* values measured at <50 μm were not considered when calculating the average modulus for simplicity. In addition to measuring the shear modulus close to the surface, it was also possible to follow-up the swelling of the hydrogels by tracking the contact point with time. This allowed the estimating of the height of the hydrogels as they swell or dissolve (*t*_sw_). The overall volumetric swelling degree *Q* at different times was estimated using the height ratio *t*_sw_/*t*_0_ assuming isotropic swelling [16]:

(2) $$Q=1+\frac{\upsilon_{sp1}}{\upsilon_{sp2}}\left(\frac{1}{w_{0}}{\left(\frac{t_{sw}}{t_{0}}\right)}^{3}-1\right)$$

where *υ_sp_*_1_ and *υ_sp_*_2_ are the specific volumes of the solvent and of the protein, respectively, and *w*_0_ is the protein weight fraction of the initial gels [11]. Note that this estimate is very approximate, for instance, because the swelling was not isotropic as shown Figure 1b,c, and the top side where indentation was performed swelled more extensively than the bottom side in contact with the glass. Moreover, these overall *Q* values are lower than the local *Q* of the swollen layer that is mechanically tested, as *Q* gradients did occur inside the gel during the early stages of swelling [16]. As imperfect as they could be considered, these overall *Q* values estimated in situ were the only data available to quantify the swelling process. Finally, note again that Equation (1) is, strictly speaking, only valid in homogeneous samples, that is, in the absence of *Q* gradients.

## 3. Results and Discussions

### 3.1. Swelling Experiments at Low NaOH Concentrations

**Figure 3:**
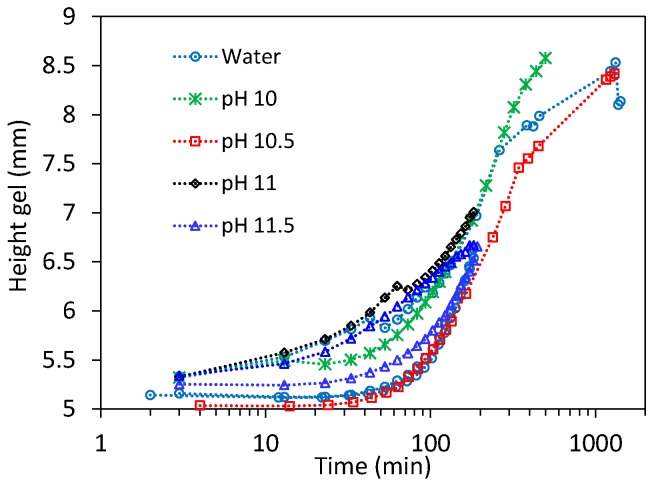
Height increase measured from the estimated contact point in swelling experiments at low alkaline pH or in water. Notice the significant variability of the dynamic swelling process between the several repeats shown, which masks the effect of the solution pH.

Figure 3 shows how in several experiments performed at pH 7–11.5, when macroscopic dissolution is typically negligible, the gels’ height increase with time from a nominal value of 5 mm. These swelling kinetics are similar to those previously determined using different techniques [16], also showing substantial variability in repeated experiments (see for example water and pH 11.5 series in Figure 3).

**Figure 4:**
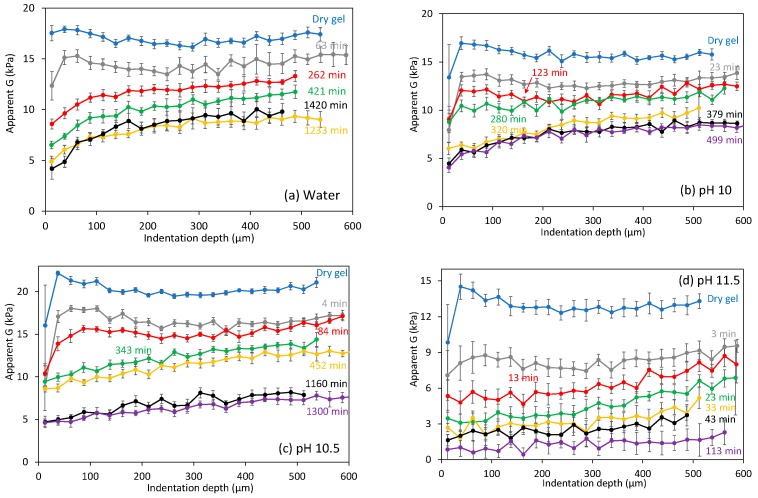
Calculated apparent shear modulus at different indentation depths in gels swollen at different conditions for different times. Dry gels refer to gel as prepared, i.e., before being submerged in solution. Points are the mean value of three indentations at different locations, error bars show the standard error. Note that as Equation (1) is only valid for homogeneous samples, *G* estimates with depth can only highlight qualitatively if substantial mechanical inhomogeneity occurs in the tested swollen layer.

At each time considered, three indentations were performed; the average shear modulus at different indentation depth is shown in Figure 4 for different swelling conditions. The initial dry gels, before being submerged in solution, sometimes showed a slightly higher modulus close to the surface, which is likely due to surface drying. The average shear modulus *G* for different dry gels tested showed a significant variability, also observed with different mechanical testing techniques. After gels were submerged in solutions, the modulus decreased substantially, but no apparent *G* gradient could be observed with the indentation depth that would suggest a *Q* gradient in the swollen layer. However, at long times, for example, >100 min, as large swelling degrees were obtained, small *G* gradients with the indentation depth were clearly apparent as shown in Figure 4. At very long times, for example, >500 min, when swelling slowed down, similar *G* profiles were obtained with time.

**Figure 5:**
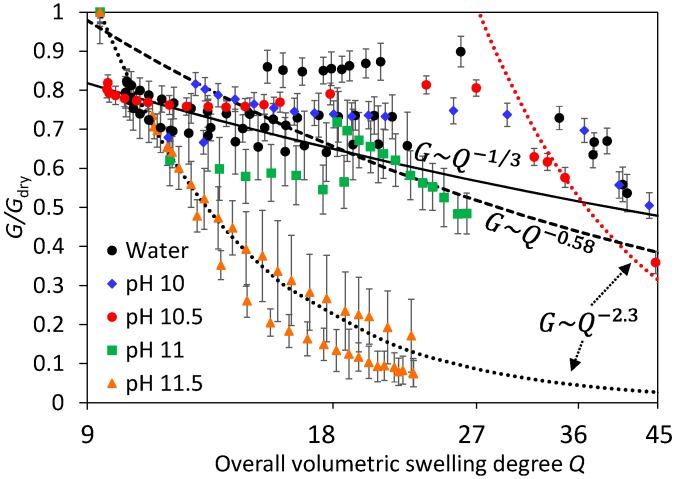
Shear modulus during swelling experiments in different solutions normalized against the initial modulus of the dry unswollen gels (before being submerged in solution). Error bars show the standard deviation of the moduli calculated between 50–500 μm indentation depths for three replicates in different locations. Lines show several theoretical scaling laws for neutral polymer gels as a comparison.

Swelling experiments at pH 11.5 were clearly different, the modulus decreases quickly and continuously up to values of the order of ~1 kPa, when intragel variability becomes relatively important. The major difference between pH 11.5 experiments and those at lower pH can be clearly observed by plotting the average shear modulus against the volumetric swelling ratio *Q*. One major limitation in these dynamic experiments is that it is currently not possible to quantify local *Q* values close to the surface. What is possible is to calculate the overall *Q* value from height measurements (Figure 3). With this important limitation in mind, which implies that surface *Q* values ought to be larger in these swelling experiments than the overall ones, *G* against *Q* is shown in Figure 5. In experiments using water or alkaline solutions at pH 10–11, it is observed a weak dependence of the modulus with the protein content that can be well represented, considering experimental variability and *Q* uncertainty, to scaling laws from the theory of rubber elasticity for flexible Gaussian chains [21,22], particularly at high crosslink densities [23]. Considering a general scaling relationship of the type *G*~*Q*^−n^, the value predicted for *n* depends on the solvent quality, at 1/3 for a Θ solvent and 0.58 for a good solvent [24], both predictions are shown in Figure 5. The reasonable agreement between the experimental data at pH 7–10 against rubber elasticity at low *Q* (<30) models suggests that chemical interactions within the gel network were stable, as in typical swelling experiments in polymer hydrogel.

Results at pH 11.5 showed, however, a much steeper decrease of *G* with *Q*, for example, *n* ~2.3. Such high exponents are expected in swollen gels at high solid contents when the mixing osmotic pressure dominates over the elastic component of the network [25,26,27]. In neutral polymers at swelling equilibrium, affine models predict that *n* = 3ν/(3ν − 1), where ν is the excluded volume exponent [28]. Values of *n* = 2.3 for good solvents (dotted lines in Figure 5) and *n* = 3 for Θ solvents are predicted and extensively observed in swollen polymer gels [29]. It is interesting that two very different scaling laws can be applied for the swelling of equal gels in the same *Q* range, yet at different pH. This is likely related to the breakdown of non-covalent interactions occurring between pH 11.2–12 [30]. The steep decline of *G* with time at pH 11.5 could be explained using rubber elasticity theory: the effective crosslinking density of the gel decreases as the gel is degraded [31]. Hence, the experimental *n* should be related to the degradation and swelling kinetics, and the value of ~2.3 is a mere coincidence. A different explanation would be to consider that the experimental *n *~2.3 is not a coincidence and is related to the previous models. That could be the case if most non-covalent interactions, the main contributors to the modulus in whey protein hydrogels [32], are quickly and extensively cleaved, thus leaving mainly the covalent disulfide crosslinks which are quite stable at pH 11.5 [30,33]. Testing these hypotheses will require better experimental data in the future, in particular measurements of the local swelling degree *Q,* as well as correct *G* measurements at different depths, for example, using CSM.

From this initial set of data, it appears that steady state *G* values were not obtained during swelling at long times (and high *Q*), apparently deviating from the weak power law behavior of rubber elasticity (see red dashed line at high *Q* in Figure 5). At such long times, *G* gradients with depth become apparent (Figure 4) when, in fact, gels would be expected to be very homogeneous. We suggest that these hydrogels were not fully stable at such high *Q* values. Most previous studies on the swelling equilibrium of whey protein hydrogels have added some NaCl in order to limit the extent of swelling [11], obtaining gels that are macroscopically stable for days. However, in the absence of salts yet in the same pH range, we have recently observed that WPI hydrogels continuously swell with time, without an apparent equilibrium value [16]. It was proposed that the gel matrix was slowly degraded, which would inhibit reaching an equilibrium *Q*, and could certainly explain the apparent large decrease of *G* with *Q* at high *Q* values and pH ≤ 11, as in pH 11.5.

### 3.2. Shrinking Experiments at High NaCl Concentrations

**Figure 6:**
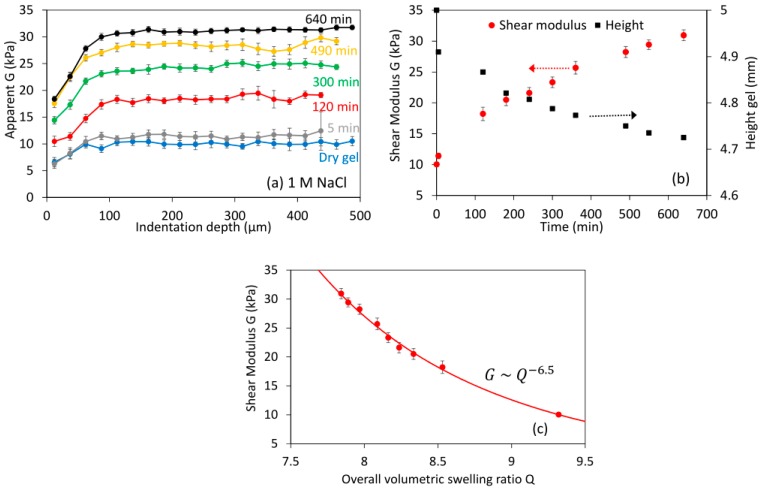
Swelling at neutral pH and at 1 M NaCl. (**a**) Shear modulus with the indentation depth at different swelling times; (**b**) average modulus (>50 μm indentation depth) and estimated gel height at different swelling times; (**c**) correlation between the shear modulus and the overall volumetric swelling ratio Q; the continuous line is the best power law fit. Error bars in (**a**) as in Figure 4, in (**b**,**c**) as in Figure 5.

In addition to swelling experiments, the new apparatus was also tested in conditions where shrinkage occurred with time. Protein hydrogels shrink when decreasing the pH close to the isoelectic point (pI) of the protein, or as performed here by increasing the salts (e.g., NaCl) concentration at a constant pH [11]. The addition of salts reduces swelling due to the minimization of the electrostatic repulsion between the proteins that drives swelling at alkaline pH. Figure 6 shows an example using 1 M NaCl at a neutral pH. Indentation experiments did not show any gradient with depth at any time (Figure 6a), despite the fact that sodium chloride concentration [NaCl] gradients should be expected within the gel. The gel height decreased slightly with time, as expected, whereas the moduli increased substantially (Figure 6b). Correlating *G* against the overall *Q* results in an exponent *n* of −6.5 (Figure 6c). Note that the local *Q* values tested during indentation should be smaller than the overall *Q* in a shrinking experiment: this would reduce the fitted *n*. Such high *n* values can be caused if high [NaCl] reduces the solvent quality, leading to the collapse of the polymer chains as *ν* decreases to 1/3 [34]. Otherwise, this large *n* value may suggest that the gel network is strengthened with time. This behavior can also be expected from the literature: weak interactions (e.g., hydrophobic) in whey protein gels and aggregates are enhanced and stabilized at high salt concentrations [35].

**Figure 7:**
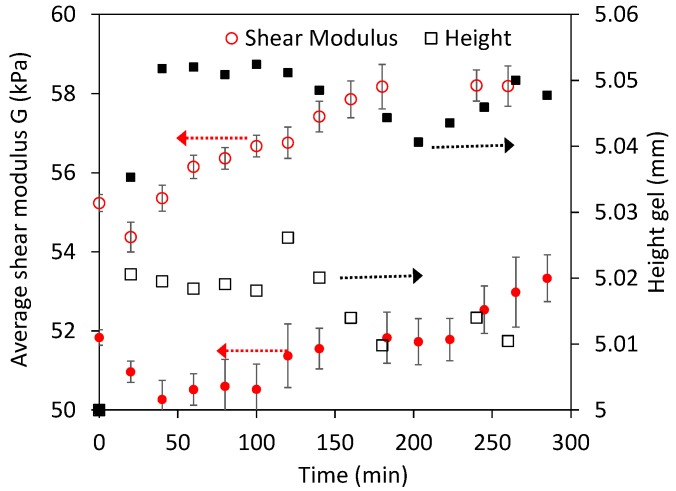
Shear modulus and height of particulate gels swollen in water (filled points) and in 0.1 M NaCl (empty points). Particulate gels were made with 0.1 M NaCl, heated at 80 °C for 1 h. Note that both the modulus and the height change little with time. Error bars are as in Figure 5.

Experiments were also performed to verify that the estimated *G* and height values were constant in conditions where little swelling should occur. Particulate WPI gels, formed for example at high salt concentrations, are known to swell very little to equilibrium at pH 7–10 [11]. Figure 7 shows the summary for the swelling of such kind of gels, swollen in water and in 0.1 M NaCl. Both the shear modulus, very constant with the indentation depth, and the height changed very little in the more than 4 h studied.

### 3.3. Dissolution Experiments at High NaOH Concentrations

**Figure 8:**
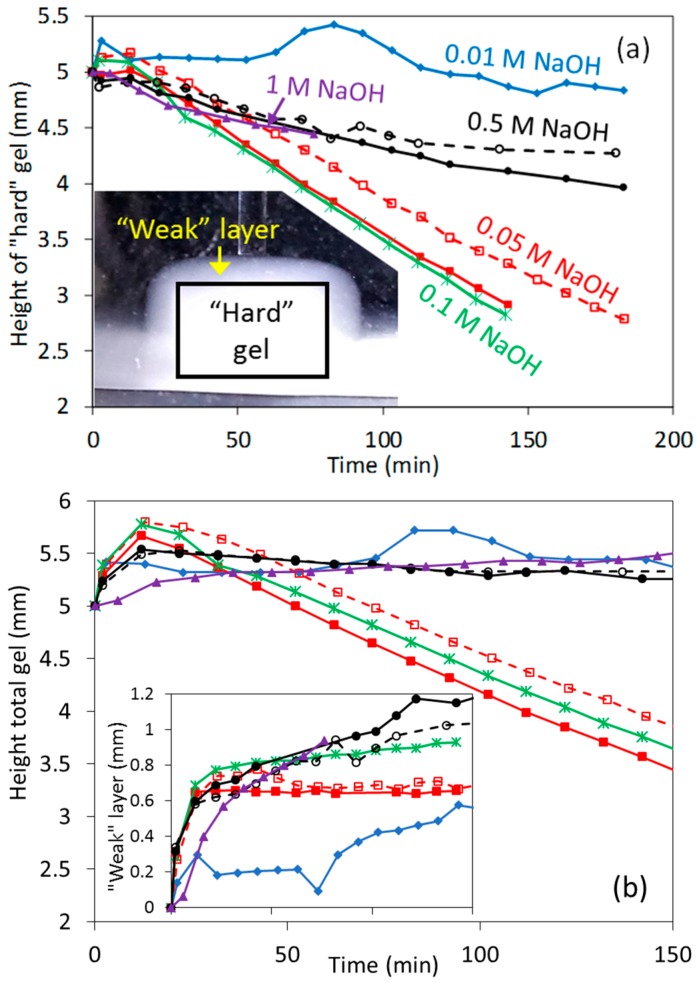
(**a**) Estimated height of the mechanically responsive gels (“hard” gels) at high (NaOH). Inset shows an example of the “weak” protein layer that forms during dissolution; (**b**) total height of gels from side microscopy measurements, including the “weak” layer shown in the inset. Empty points and dashed lines are used for repeated experiments.

Experiments were then performed to study alkaline dissolution in a wide range of concentrations. It is well known from the literature that the dissolution of whey protein gels at low [NaOH] (e.g., 0.01 M), as well as at very high [NaOH] (e.g., 0.5–1 M), proceeds slowly at ~20 °C; whereas in intermediate [NaOH] (e.g., 0.05–0.1 M) it shall proceed quite quickly [3,33]. This expected dissolution behavior was well reproduced in height measurements (Figure 8), as the gels height decreased very slowly at 0.01, 0.5 and 1 M NaOH compared to experiments at 0.05 and 0.1 M NaOH.

Height measurements at these [NaOH], when dissolution occurs, were different than in the previous swelling experiments. Two different heights were actually measured. The first one was that observed by the side microscope (Figure 1), referred as the total height (Figure 8b). However, no mechanical response was observed when indenting this layer until much deeper, resulting in a second height for the “hard” gel from indentation measurements. We refer, for simplicity, to the “weak” layer that is located in between the boundary observed by microscopy and by indentation. The Figure 8b inset shows that this weak layer could be quite substantial, for example, 0.5–1 mm, and was found to reach a constant value at 0.05 and 0.1 M NaOH, whereas at the other [NaOH] tested it continuously increased with time. Because of this, the decrease with time during dissolution of the total and “hard” heights gels were very similar at 0.05 and 0.1 M NaOH; whereas at the other [NaOH] the slight decrease in the “hard” height was compensated by the increase of the “weak” layer, resulting that the total height barely changed with time.

It is calculated from Figure 8 an average dissolution speed of 15.8 μm/min for both the 0.05 and 0.1 M NaOH experiments, resulting in an estimated dissolution rate of ~0.038 g protein m^−2^ s ^−1^. This value is reasonable considering previous dissolution studies at room temperature [3,33], although a higher rate was expected at 0.1 M NaOH. The dissolution speed calculated from the “hard” gel height at 0.5 and 1 M NaOH was ~2.3 μm/min at <100 min, which corresponds to an average dissolution rate of 0.0055 g protein m^−2^ s ^−1^, and is also in good agreement with the literature [3].

**Figure 9:**
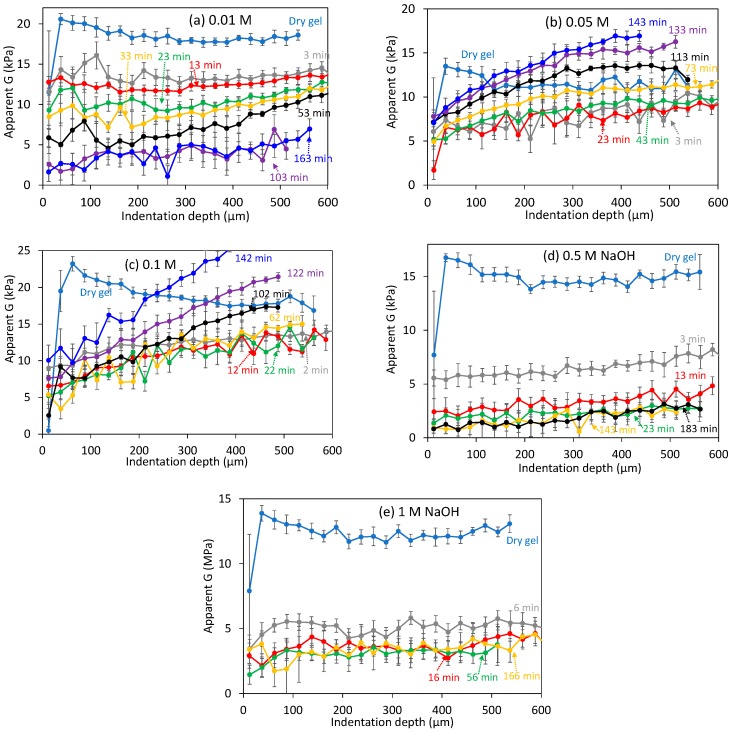
Calculated shear modulus at different indentation depths in (NaOH) typical of dissolution experiments. Note that as Equation (1) is only valid for homogeneous samples, *G* estimates with depth can only highlight qualitatively if substantial mechanical inhomogeneity occurs in the tested swollen layer. Error bars are as in Figure 4.

The calculated shear modulus at different indentation depths is shown in Figure 9 at several representative times, but only for the “hard” mechanically responsive part of the gels. Results for 0.01 M NaOH (e.g., pH 12), Figure 9a, were similar to those at pH 11.5 (Figure 4d). Small *G* gradients were observed at long times when the average *G* values had already significantly decreased. Profiles for the 0.05 and 0.1 M NaOH experiments were markedly different: they decreased quickly from the initial dry conditions, showing a very clear gradient with the indentation depth unlike seen before. In addition, these profiles were very constant, typically up to <70 min, as shown in Figure 9b,c. At longer times, the shear moduli actually increased with time, but still showing very clear gradients. In fact, moduli higher than the values tested in the initial dry conditions were observed, although these values were still reasonable considering the variability of gels. Experiments at 0.5 and 1 M NaOH, Figure 9d,e, resulted in very different modulus profiles: *G* decreased very quickly, without apparent gradients with the indentation depth, until very small *G* values were obtained, which became constant with time.

**Figure 10:**
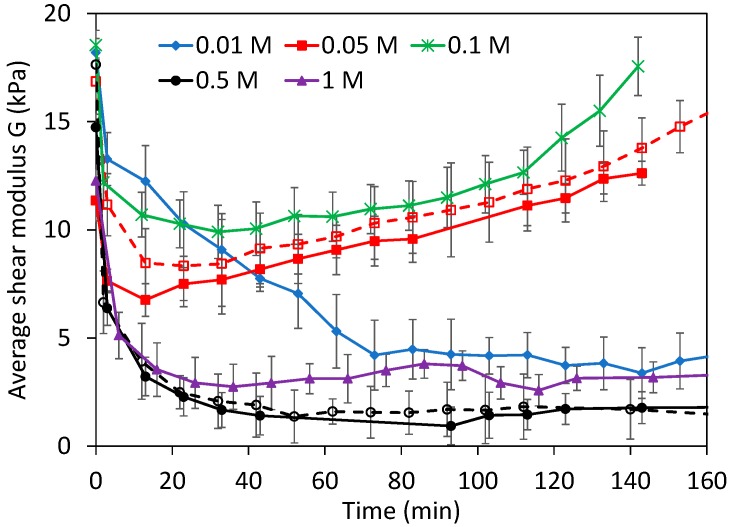
Shear modulus in WPI gels undergoing dissolution at different (NaOH). Moduli were calculated as the average between 50–500 μm indentation depth. Empty points and dashed lines are used for repeated experiments. Error bars are as in Figure 5.

Dynamic effects are better observed in Figure 10 considering the average *G*. Experiments at 0.05 and 0.1 M NaOH, when dissolution is faster, clearly presented much higher moduli than when dissolution was slow. Interestingly, in all experiments where the average moduli are constant with time, the dissolution rates are also known to be constant [3]. Constant dissolution rates imply that the system has reached a steady state, with should also be observed on the swollen/degraded hydrogel next to the solution boundary tested here, for example, the local *Q* profile, the degree of chemical degradation, and so on. The constant moduli should be the consequence of the steady-state swollen layer, despite the fact extensive dissolution occurs. The depths indented at <500 μm here were relevant to test the swollen layer because the constant penetration depth of the NaOH, where the pH > 9.8, was around ~1 mm [36]. From these initial experiments, we can conclude an apparently contradicting observation: the swollen layer is harder in conditions where chemical dissolution is faster.

The moduli increase at >100 min in experiments at 0.05 and 0.1 M NaOH could possibly be an artifact. Note that at those times the gels have already dissolved extensively (Figure 1c), hence the gel height and diameter were both much smaller. Substrate effects in indentation, artificially increasing the calculated *G,* become important due to the correction factor Π(*R*/*t*_0_*,ν_P_*) in Equation (1). However, the moduli is still observed to increase in Figure 10 despite the data had been corrected due to the reduction of *t*_0_. Another reason may be related to the fact that, after extensive dissolution, a typical ~500 μm indentation would result in large strains where the Equation (1) may not be valid.

### 3.4. Non-Elastic Deformation

**Figure 11:**
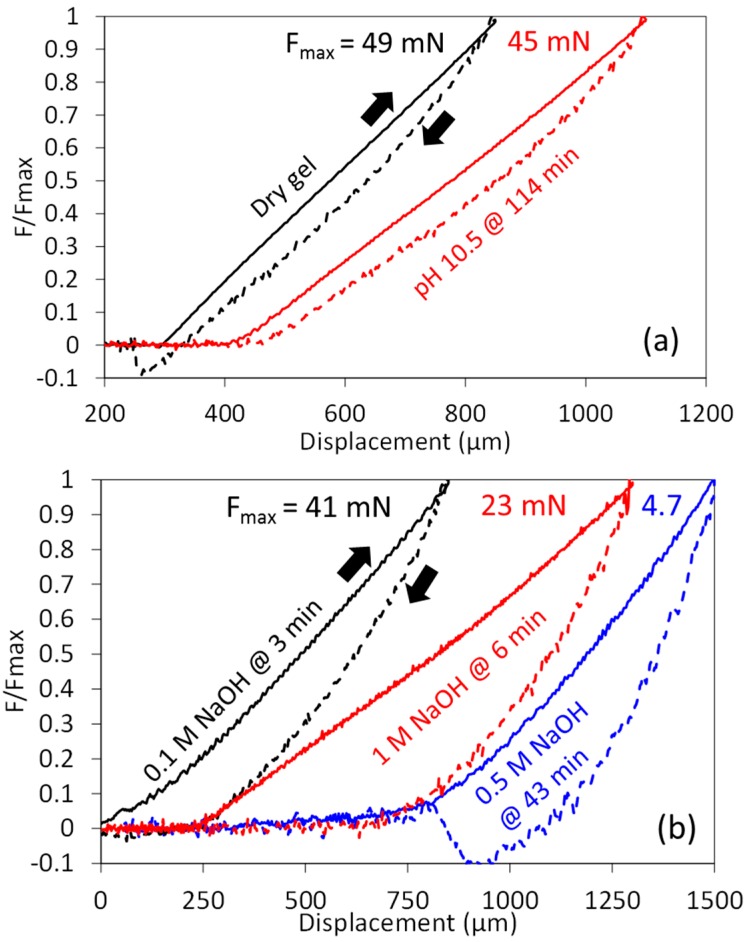
Typical loading-unloading indentations, continuous and dashed lines respectively. (**a**) Gels tested dry or swollen at low alkaline pH; (**b**) gels undergoing dissolution at high [NaOH]. For clarity, the force is normalized with the maximum force, shown at the top, and some data has been shifted horizontally to avoid overlapping.

Indentation experiments can also provide information of the non-elastic nature of hydrogels by studying the hysteresis of loading-unloading experiments. Figure 11a shows typical examples of dry gels and of gels at low alkaline pH when only swelling occurs. The hysteresis was small and it remained mostly the same during swelling. The elastic recovery was high in all cases, at ~92%. As indentation experiments were performed at 60 μm/s, ~10 s was required for each unloading and unloading steps. In such time frames, it has been shown that the force relaxes ~20% due to a combination of poro- and viscoelastic effects [37], and such value is reasonable in the hysteresis seen here. Note, then, that the modulus calculated during the loading step is not the instantaneous modulus, namely, that which would be obtained at very fast loading speeds.

Experiments at high [NaOH] yielded very different results, as shown in Figure 11b. Even in the earliest measurements performed, typically after ~3 min of being submerged in solution, the hysteresis was substantially larger than before. The elastic recovery also decreased markedly, for the data shown in Figure 11b it is ~70% at 0.1 M NaOH, and ~56% at 0.5–1 M NaOH. Clearly, this is related to the destruction of the elastic gel network by the alkali, and this information should be useful to quantify the destruction of the chemical interactions from a mechanical point of view. Finally, adhesion between the indenter and the hydrogel was also observed in many measurements, particularly during dissolution, as inferred from the negative force values during the unloading step. Adhesion forces were relatively large for the dissolution experiments in 0.5 M NaOH, as shown in Figure 11b, due to the very small shear modulus.

## 4. Conclusions

We presented in this study a novel use of microindentation in order to assess the mechanical characterization of hydrogels under reactive conditions. An apparent shear modulus was estimated at different indentation depths in order to provide insights of the swollen layer next to the interface; proper understanding would require extensive finite element simulations with heterogeneous materials. The results showed, however, that very few *G* gradients could be observed during swelling-only experiments, probably due to the weak dependence of *G* with the swelling degree *Q*. Gradients with depth are, however, observed in conditions where degradation of the gel network occurs. Chemical degradation, or strengthening, of hydrogels is apparent by steep power-law dependencies. The mechanical analysis of hydrogels undergoing dissolution is consistent with the literature, providing the first evidence that the mechanics of the swollen layer are constant when the dissolution rate is also constant. In addition, we have confirmed that at very high [NaOH] chemical interactions are extensively destroyed, quickly yielding very low and constant moduli, yet dissolution proceeds very slowly. In such conditions, mechanical cleaning of hydrogels would be most effective. Visualization of dissolution has shown the existence of a stagnant protein layer, which offered no mechanical resistance to indentation, but was not rinsed away by the fluid. Therefore, proteins aggregates are still expected to be crosslinked. Analysis of the hysteresis in cycles can further provide information on the non-elastic nature of the swollen layer due to chemical degradation. This initial study shows the great potential of microindentation to understand hydrogel dissolution mechanisms under reactive conditions.
